# Morphological Characterization of Bushy Cells and Their Inputs in the Laboratory Mouse (*Mus musculus*) Anteroventral Cochlear Nucleus

**DOI:** 10.1371/journal.pone.0073308

**Published:** 2013-08-26

**Authors:** Amanda M. Lauer, Catherine J. Connelly, Heather Graham, David K. Ryugo

**Affiliations:** 1 Department of Otolaryngology-HNS, Johns Hopkins University, Baltimore, MD, USA; 2 Hearing Research Unit, Garvan Institute of Medical Research, Darlinghurst, New South Wales, Australia; 3 School of Medical Sciences, University of New South Wales, Kensington, New South Wales, Australia; University of Southern California, United States of America

## Abstract

Spherical and globular bushy cells of the AVCN receive huge auditory nerve endings specialized for high fidelity neural transmission in response to acoustic events. Recent studies in mice and other rodent species suggest that the distinction between bushy cell subtypes is not always straightforward. We conducted a systematic investigation of mouse bushy cells along the rostral-caudal axis in an effort to understand the morphological variation that gives rise to reported response properties in mice. We combined quantitative light and electron microscopy to investigate variations in cell morphology, immunostaining, and the distribution of primary and non-primary synaptic inputs along the rostral-caudal axis. Overall, large regional differences in bushy cell characteristics were not found; however, rostral bushy cells received a different complement of axosomatic input compared to caudal bushy cells. The percentage of primary auditory nerve terminals was larger in caudal AVCN, whereas non-primary excitatory and inhibitory inputs were more common in rostral AVCN. Other ultrastructural characteristics of primary auditory nerve inputs were similar across the rostral and caudal AVCN. Cross sectional area, postsynaptic density length and curvature, and mitochondrial volume fraction were similar for axosomatic auditory nerve terminals, although rostral auditory nerve terminals contained a greater concentration of synaptic vesicles near the postsynaptic densities. These data demonstrate regional differences in synaptic organization of inputs to mouse bushy cells rather than the morphological characteristic of the cells themselves.

## Introduction

All input from the auditory nerve terminates in the cochlear nucleus of the brainstem [1], which is grossly divided into ventral and dorsal divisions. Further divisions of the ventral cochlear nucleus into anterior and posterior subdivisions have been made on the basis of the bifurcation zone of the auditory nerve from which ascending and descending branches project [1]; however, subdivisions based on cytoarchitectonic descriptions have also been proposed [2,3].

Detailed characterization of the cell types within each division is fundamental for understanding structure-function relationships in auditory pathways. Such characterizations must be done on a species by species basis, since structural and physiological variations often reflect the auditory specializations that evolved to optimize an organism’s survival within its ecological niche.

In addition to the obvious physical differences between, for example, mice and cats, what is critical to our analyses is the difference in their audible frequency range. Whereas cats hear down to around 100 Hz, mice do not hear much below 1000-2000 Hz and their range of sensitivity extends up to 80-100 kHz [4,5]. This relative lack of low frequency hearing has been linked to a reduction in pathways that use associated cues [6,7]. The associations of some bushy cell subtypes with low frequency hearing [8,9] suggests that there could be complications when comparing bushy cells across species having different natural histories.

Principal cells of the anteroventral cochlear nucleus (AVCN) are typically classified according to cytologic criteria established for the cat [8,10], a low-frequency hearing species relative to the mouse. Two types of AVCN principal cells, the spherical and globular bushy cells (SBCs and GBCs), receive huge auditory nerve endings, called “endbulbs of Held” and “modified endbulbs,” specialized for precise temporal firing [11,12]. These high fidelity synapses are essential for coupling neural activity to acoustic events, and are involved in auditory processing tasks for which temporal precision is critical, such as encoding amplitude modulation [13,14] and sound localization cues [15,16].

Some authors have noted that there are few SBCs in the rostral AVCN of mice [2], and that they differ in appearance compared to SBCs found in other mammalian species [17]. Specifically, the somata of mouse SBCs are smaller, have more ambiguous shapes, and frequently lack a perinuclear necklace of Nissl substance. Recent physiological studies in mice have called into question the clear distinction between SBCs and GBCs in mice, suggesting instead a sort of continuum of response properties determined by the inputs to the cell with exemplars of both response types at either end of the spectrum [18,19]. Indeed, there are examples of classic SBCs and GBCs in mice [2,20], but a detailed quantitative characterization of regional differences in bushy cell morphology in the mouse is lacking.

Differences in synaptic inputs may be most predictive of functional characteristics of the cell. A wide variety of response types may be associated with AVCN cells of a particular morphology [21,22]. Mouse bushy cells may be parsed into subtypes based on the patterns of inputs and projections rather than distinct cell body morphology. Physiological studies have characterized bushy cells into two subtypes based on the distribution of response properties [23,24]. Cao and Oertel (2010) estimate that mouse SBCs receive input from 1–2 auditory nerve fibers and GBCs receive input from 3 or more auditory nerve fibers, suggesting that there is at least some morphological distinction in inputs. Response properties of cochlear nucleus neurons are likely determined by the size, number, type, and site of auditory nerve inputs as well as presynaptic and postsynaptic active zone characteristics, and intrinsic membrane properties [25]. Inhibitory inputs may also affect bushy cell responses, though these effects may differ across neurons with bushy cell-like responses [26,27]. Modulatory influences by other types of inputs (i.e., cholinergic, serotinergic) to bushy cells have not been clearly characterized *in vivo*; however, experiments conducted in mouse brainstem slice preparations indicate that these types of influences are weak [18,28].

We conducted a systematic investigation of mouse bushy cells and their synaptic inputs along the rostral-caudal axis of AVCN using light and electron microscopy and immunohistochemistry in an effort to address some of the issues of bushy cell subtypes. We determined that bushy cells could not be unambiguously identified as spherical or globular based solely on cell body characteristics. We found regional differences in synaptic organization for bushy cells in caudal and rostral AVCN, which relate to physiological response characteristics. Collectively, the data suggest that bushy cell response properties may be largely determined by afferent inputs.

## Materials and Methods

### Ethics Statement

All procedures were approved and performed in accordance with the Guide for the Care and Use of Laboratory Animals and the Johns Hopkins University Animal Care and Use Committee (Animal Welfare Assurance # A3272-01) and the Garvan/University of New South Wales Animal Ethics Committee. All surgical procedures were carried out with anesthesia as described below, and all efforts were made to minimize suffering.

### Subjects

Twenty adult normal-hearing male and female CBA/CaJ mice were obtained from Jackson Laboratories (Bar Harbor, ME) or bred in an institutional rodent facility. Tissue was harvested from subjects at 2-3 months of age. Animals were housed in standard shoebox cages with ad libitum access to food and water and checked twice per day for general health status. Tissue from 3 mice was processed for Nissl staining and light microscopy, tissue from 8 mice was processed for immunohistochemistry, and tissue from 5 mice was processed for electron microscopy (2 were also processed for immunohistochemistry). Six mice were used for neuronal tracer injections.

### General tissue preparation

The animals were given an overdose of 50mg/ml sodium pentobarbital (0.2cc per 20g body weight i.p.) and transcardially perfused with 3 ml of 1% NaNO_2_ solution followed by 60 ml of either 4% paraformaldehyde (for immunohistochemistry and light microscopy) or a mixture of 2% glutaraldehyde and 2% paraformaldehyde (for electron microscopy). The skull bones were partially removed and the brains were post-fixed overnight in the perfusion solution. The following day, brains were dissected from the skull, blocked, embedded in gel albumin, and sectioned with a vibrating microtome. Sections were then processed for light or electron microscopy as described below.

### Basic light microscopy

50 µm thick sections were mounted onto glass slides and air-dried before being stained with cresyl violet, dehydrated in graded alcohols, and cover-slipped. Sections throughout the AVCN were photographed with 10x and 40x objective lenses for offline analysis. The position relative to the caudal pole of AVCN was recorded. Ovoid or round cell bodies with a diameter greater than 10 µm and their nuclear silhouettes visible in the 40x images were traced in *Adobe Photoshop* if the structure had clear boundaries, a visible nucleus, and at least one nucleolus present. Multipolar cells with triangular or irregular cell body shapes were excluded, as were small cells with a diameter of less than 10 µm. The most extreme rostral sections through AVCN were not included in the analysis because few bushy cells were visible in these sections.

Cross sectional area, roundness, and aspect ratio (long axis/short axis) were calculated for each cell trace using NIH *ImageJ* software. Nucleus cross sectional area, percentage of cell body area, and nucleus position were also measured using ImageJ. The eccentricity of nuclei within the cell bodies was calculated using the following equation: √(x centroid of cell – x centroid of nucleus) + (y centroid of cell – y centroid of nucleus)^2^.

### Immunohistochemistry

Tissue was sectioned in the coronal plane at 50µm and immunohistochemically stained for vesicular glutamate transporter 1 (VGlut1) as previously described [29]. Sections were developed with the addition of 0.4% nickel ammonium sulfate to the 3,3’-diaminobenzadine solution, to give the reaction product a high contrast, dark purple color. VGlut1 is a marker for glutamatergic nerve terminals in the cochlear nucleus [30–32], and therefore stains excitatory terminals. Negative controls (processed with buffer only during primary antibody incubation) were run on several sections. Positive controls for VGlut1 reactivity were cerebellum and cortex. Sections were counterstained using cresyl violet and bushy cell identification was guided by cytologic criteria [2,8,33]. Bushy cells were photographed with a Nikon Eclipse E600 microscope fitted with a 100x oil immersion lens or a Zeiss 710 confocal microscope fitted with a 40x lens. Sections designated for electron microscopy were processed and photographed as described below.

Every other 50µm thick tissue section of VGlut1 stained AVCN tissue was selected for quantitative analysis of excitatory nerve boutons terminating onto bushy cells, beginning at the auditory nerve root. Positively stained VGlut1 puncta surrounding the equator of bushy cells were outlined using *Neurolucida*. Total number and average area of VGlut1 positive puncta were calculated per cell.

### Electron Microscopy

75 µm sections were post-fixed with osmium tetroxide in 0.1M s-collidine buffer (pH 7.4), rinsed in 0.1M maleate buffer (pH 5.2), stained with 1% uranyl acetate in 0.1M maleate buffer (pH 6.0), and rinsed again in maleate buffer. Sections were dehydrated with graded alcohols and propylene oxide, infiltrated with Polybed 812, flat-embedded, and baked at 60°C for 1-2 days. Regions containing bushy cells were cut from selected sections and embedded in BEEM capsules for ultrathin sectioning. Ultrathin sections from each block were cut at 75 nm, mounted on slotted grids, and stained with uranyl acetate and lead citrate. Random sections were analyzed using a Hitachi H7600 or H7650 transmission electron microscope. Bushy cells in which the cell body, nucleus, and nucleolus were visible were photographed at 3000x or 5000x. Apposing synaptic terminals were photographed at high magnification (15,000x). Black and white levels were adjusted (entire image) to normalize contrast and brightness as much as possible across images.

Structural features were traced using *Adobe Photoshop* and a Wacom Cintiq interactive drawing tablet. Traced features included cross-sectional somatic (silhouette) area, mitochondria, and nuclei. Synaptic profile area was traced for all types of terminals. Each section through a synaptic terminal, also referred to as an ending or profile, was classified as a primary auditory nerve ending (large round vesicles, asymmetric postsynaptic densities), or non-primary ending. Profiles with small round vesicles and asymmetric postsynaptic densities are excitatory, but originate from sources other than the auditory nerve. Profiles with pleomorphic or flat vesicles and symmetric postsynaptic densities are inhibitory. These criteria are based on work by Tolbert and Morest [34] and have been used by others to classify terminals [35,36]. Additional synaptic details were traced for primary auditory nerve terminals with clearly visible postsynaptic densities (PSDs), including PSD length and curvature, mitochondrial volume fraction [37] and the number of synaptic vesicles (SVs) within 500 nm of the PSD [37,38]. Features were measured using *ImageJ* software. Data were entered into Excel spreadsheets and statistically analyzed using Kaleidagraph software. Non-parametric Wilcoxin Mann–Whitney U tests were used to test for statistical differences.

### Neuronal tracer injections

Mice were anesthetized with 1-2% isoflurane and secured in a stereotaxic frame (Stoelting, Wood Dale, IL). A craniotomy overlying the inferior colliculus and/or cerebellum was made approximately 0.5 mm lateral and 6.0 mm caudal to Bregma. All surgical procedures were carried out using aseptic techniques. A glass micropipette (~15µm inner diameter; impedance 2-6 MΩ), was filled with biotinylated dextran amine (MW10,000; D-1817; Molecular Probes) diluted to 10% w/v in a solution of 0.05M Tris buffer, pH 7.6, and 0.15M KCl, and attached to a micromanipulator. To approach the medial nucleus of the trapezoid body (MNTB), the electrode was inserted at an angle 10^°^ caudal off of the vertical axis using a motorized hydraulic micromanipulator (2650; Kopf Instruments, Tujunga, CA). Broadband noise was presented to the animal from a loudspeaker as the electrode was advanced into the brain. Arrival into the MNTB was marked by the presence of sound-evoked spike discharges. Dextran-amine tracer was injected iontophoretically using a high voltage, constant current source (CS 3; Midgard/Stoelting) set at 5 µA of positive current (50% duty cycle) for 6-10 min when a sound-evoked response was recorded in a region calculated to be at the level of the MNTB. After a rest period of 5 min. the pipette was withdrawn. Upon conclusion of the experiment the craniotomy was covered with bonewax, and the animal allowed to recover.

Animals were deeply anesthetized with a lethal dose of sodium pentobarbital (100 mg/kg, IP) and perfused transcardially with 4% paraformaldehyde in 0.1M phosphate buffer (PB), pH 7.3 two weeks following dye injection. The brainstem was dissected from the skull and postfixed overnight in 4% paraformaldehyde. Embedding and sectioning was performed as described above. Sections were histologically processed using standard methods involving avidin-biotin (Vectstain ABC Systems, Vector Labs, Burlingame, CA) with nickel-enhanced diaminobenzidine (Doucet and Ryugo, 2003). One injection site was located on the midline of the trapezoid body, and two injections were located in or around the contralateral MNTB. One injection site was dorsal to the MNTB and two were apparently outside the ventral border of the brainstem. The animal with the successful midline injection site was chosen for analysis. BDA-stained structures appear black when viewed with a light microscope. Sections containing well-stained neurons in the cochlear nucleus were selected for processing for electron microscopy as described above. Labeled cells were photographed in flat sections and then several cells were selected for further processing and photographing with the electron microscope. Labeled dendrites and their synaptic contacts were also photographed.

## Results

### Cell body shape and nucleus eccentricity in anterior and posterior AVCN

**Figure 1:**
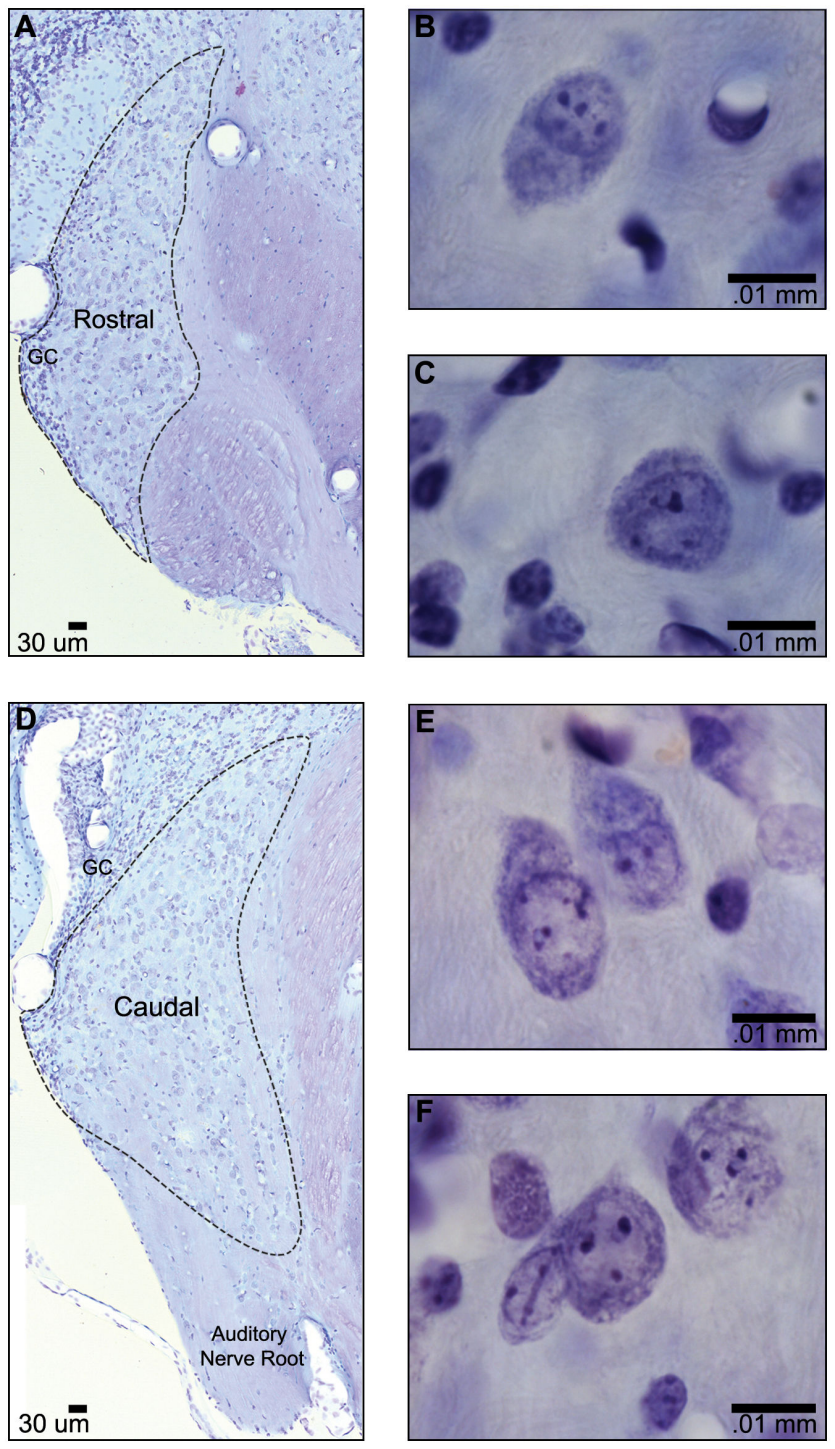
Photomicrographs of Nissl-stained AVCN. (**A**) Section through rostral AVCN at low magnification. Granule cells (GC) are present along the lateral edge. (**D**) Section through caudal AVCN at low magnification. The lateral and dorsal edges of the nucleus are bounded by GCs, and the auditory nerve (AN) root is visible ventrally. Higher magnification images of large round and ovoid bushy cells of rostral AVCN (B, C) and caudal AVCN (D, E) demonstrate that cells display both centric and eccentric nuclei.

Figure 1 shows low and high magnification images of Nissl-stained cell bodies observed in rostral AVCN (A-C) and caudal AVCN (D–F). Cells with round or ovoid shapes, presumed to be bushy cells, were most common in the core of the nucleus, whereas small, darkly stained granule cells (GC) were found along the lateral and dorsal borders of AVCN. Irregularly shaped multipolar cells were occasionally observed in the core of the nucleus. Higher magnification images revealed ovoid (Figure 1 B, E) or round (Figure 1 C, F) cell body silhouettes in both rostral AVCN and caudal AVCN. Similarly, both centric (Figure 1 C, F) and eccentric (Figure 1 B, E) nuclei were observed in both rostral and caudal AVCN.

Correlation coefficients (Pearson) were calculated to investigate the relationship between cell body cross-sectional area, aspect ratio, and roundness as a function of position along the rostral-caudal axis relative to the caudal-most pole of AVCN. A total of 2,383 cells were analyzed. There were very weak correlations between cross-sectional area (r=0.12), aspect ratio (r=0.077), and cell body roundness (r=0.075) and position along the rostral-caudal axis. Correlation coefficients were also calculated to investigate the relationship between position along the rostral-caudal axis and nucleus cross-sectional area, percentage of cell body area occupied by the nucleus, and nucleus eccentricity. There were again very weak correlations between nuclear cross-sectional area (r=0.064), percentage of cell body area (r=0.071), and eccentricity (r=0.061) with respect to position in AVCN.

### VGlut1-positive auditory nerve terminals in AVCN

**Figure 2:**
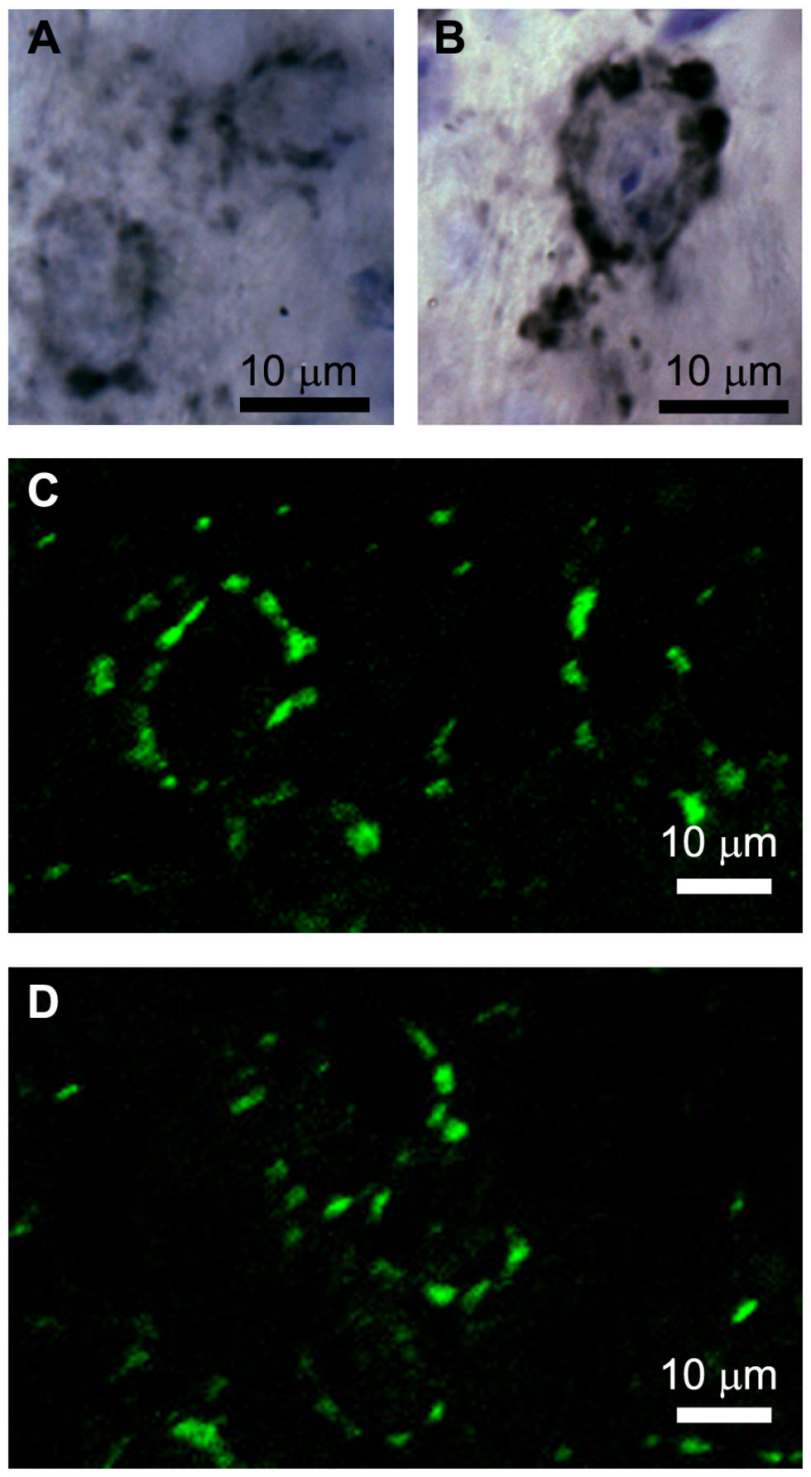
Photomicrographs of Vglut1-positive auditory nerve endbulbs and bouton terminals contacting bushy cells of rostral AVCN (A, C) and caudal AVCN (B, D). Some small Vglut1-positive terminals are also visible in the neuropil. The cells in A and B were counterstained with cresyl violet to visualize cell bodies and photographed using traditional transmitted light microscopy. The cell bodies in C and D were not labeled, but their locations are inferred on the basis of circular and ovoid rings of Vglut1-positive terminals.

Both small and large auditory nerve terminals were labeled with antibodies against VGlut1 (Figure 2 A–D). Bushy cells were contacted by axosomatic VGlut1-positive puncta/terminals covering most of the perimeter of the cell body in both rostral (Figure 2 A, C) and caudal (Figure 2 B, D) AVCN. VGlut1-positive terminals were also observed in the neuropil, presumably contacting dendrites of bushy and multipolar cells. Analysis of VGlut1-positive terminals contacting 86 bushy cells revealed no significant correlations between position relative to caudal AVCN pole and the number of VGlut1-positive puncta contacting the cell body (r=0.224), the total area of the end bulb profiles per cell body (r=0.203), or the mean area of each VGlut1-positive puncta (r=0.029).

### Ultrastructure of bushy cells and their synaptic inputs

**Figure 3:**
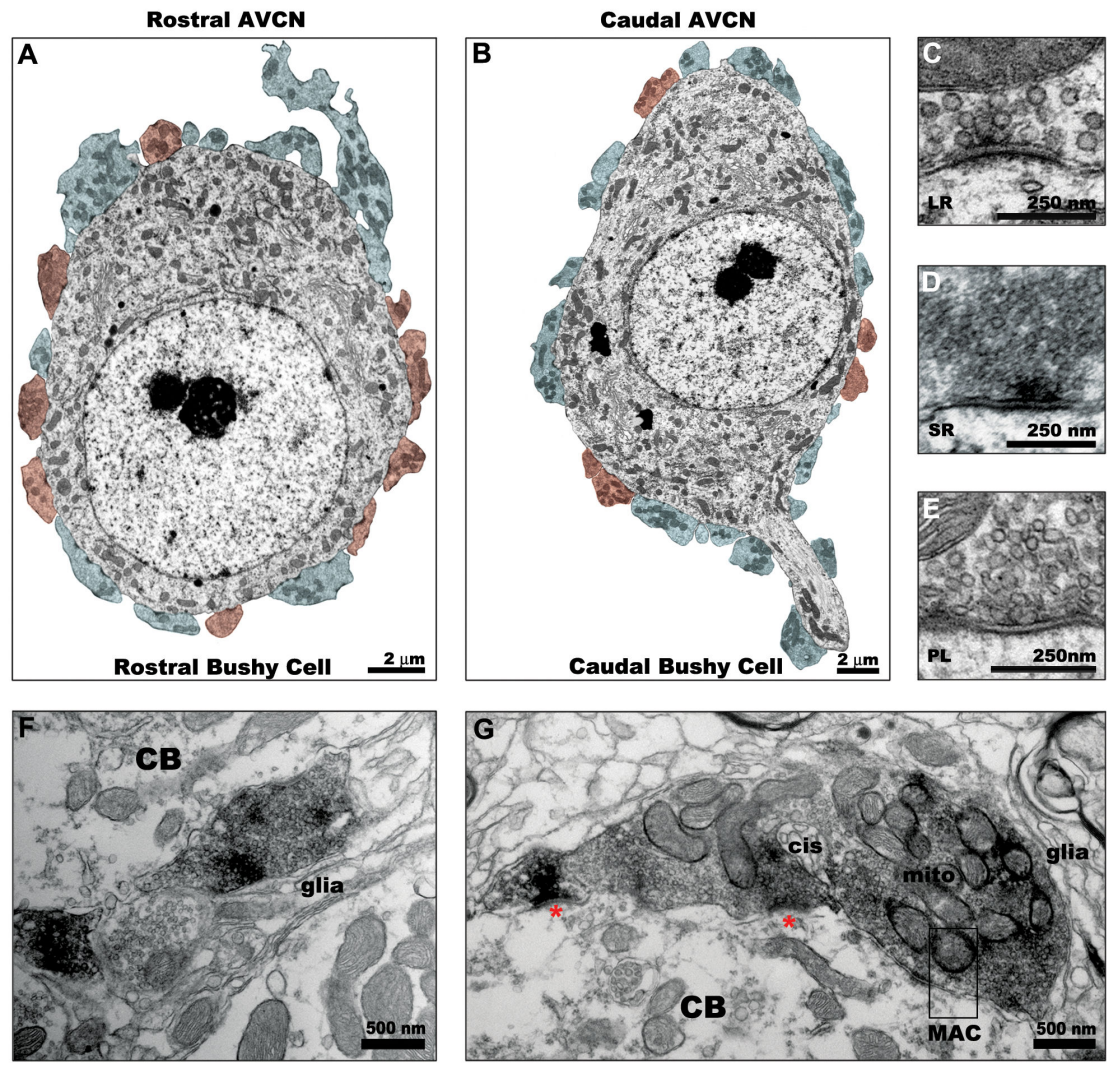
Electronmicrographs of bushy cell bodies and their synaptic contacts in the AVCN. Bushy cells in both rostral AVCN (A) and caudal AVCN (B) receive numerous auditory nerve terminals (blue). Non-primary terminals originating from sources other than the auditory nerve (red) are more commonly observed contacting bushy cells in rostral AVCN compared to caudal AVCN. Three types of terminals form synapses with bushy cell bodies, characterized by (C) large round (LR) synaptic vesicles (SVs) and curved, asymmetric postsynaptic densities (PSDs); (D) small round (SR) SVs with flat, more symmetric PSDs, and pleomorphic (PL) SVs with symmetric PSDs of varying curvature. Terminals with LR SVs are VGLUT1-positive (F, G), consistent with their origin from the auditory nerve and are either bouton-like (F) or large endings with curved PSDs (asterisks), mitochondrial clusters, intermembramous cisternae (cis), and mitochondrial adherens complexes (MAC) characteristic of end bulb synapses (G).

Ultrastructural features of bushy cell bodies and their synaptic inputs were analyzed for 12 cells in rostral AVCN and 12 cells observed in caudal AVCN. Examples of bushy cells from both regions are shown in Figure 3 A and B. The range, median, and p-values for ultrastructural features and rostral-caudal comparisons are summarized in Table 1. Confirming that the selection of cells sampled were not biased toward the prototypical round cell body shape in rostral AVCN and the oblong shape in caudal AVCN, there was no statistical difference in cell body aspect ratio between groups of cells (Wilcoxin Mann-Whitney U; p=0.843) and no gross differences in the structure or distribution of organelles within the cell bodies were observed. The pale nuclei were typically round or contained only slight invaginations, and the prominent “Nissl necklace” described in cats was less pronounced in mice. The cytoplasm was filled with mitochondria, smooth and rough endoplasmic reticulum, and Golgi apparatuses. Multivesicular bodies and lysosomes or lipofuscin granules were occasionally observed. Bushy cells of rostral AVCN showed slightly more mitochondria per 10 µm^2^ compared to caudal AVCN bushy cells (p=0.001), and the average (per cell) mitochondria size was larger in rostral AVCN (p=0.002) Bonferonni correction for multiple comparisons were applied to determine significant p-values.

**Table 1:** Comparison of ultrastructural characteristics of bushy cells and primary auditory nerve terminals of the rostral and caudal mouse AVCN.

**Parameter**		**Range (max/min)**	**Median**	**p-value**
*Cell Body*				
Aspect ratio (long vs. short axis)	aAVCN	1.565/1.060	1.282	0.843
	pAVCN	1.576/1.090	1.298	
Number mitochondria per 10 µm^2^	aAVCN	1.160/0.769	.980	0.001*
	pAVCN	1.047/0.552	.747	
Average mitochondria size (per cell)	aAVCN	0.124/0.055	.099	0.002*
	pAVCN	0.146/0.093	.113	
Number of terminals per 10 µm (perimeter)	aAVCN	4.896/2.652	3.80	0.114
	pAVCN	4.270/2.633	2.20	
Percent perimeter apposed by terminals	aAVCN	87.792/55.037	73.614	p<0.001*
	pAVCN	75.073/48.90	58.991	
Percent primary terminals	aAVCN	68.421/30.0	46.310	0.0079*
	pAVCN	89.470/34.780	68.586	
*Primary Auditory Nerve Terminals*				
Primary profile cross sectional area (µm^2^)	aAVCN	12.379/0.590	2.204	0.085
	pAVCN	15.983/0.295	1.323	
PSD length (µm)	aAVCN	0.494/0.159	0.314	0.502
	pAVCN	0.586/0.169	0.330	
PSD curvature	aAVCN	0.487/0.098	0.249	0.903
	pAVCN	0.799/0.110	0.233	
Percent mitochondria area	aAVCN	52.53/0	20.410	0.396
	pAVCN	34.201/0	19.252	
Number of SVs per µm^2^ within 0.5 µm of PSD	aAVCN	271.277/54.054	131.098	p<0.001*
	pAVCN	180.064/29.101	105.456	

^*^ statistically significant , p<0.0083

Three types of axosomatic synapses were observed in both rostral and caudal AVCN. Primary synapses with large round (LR) synaptic vesicles and curved, asymmetric postsynaptic densities (Figure 3 C) characteristic of auditory nerve synapses described in other species were most abundant. This type of synapse was VGlut1-positive (Figure 3, F-G). Secondary synapses with either small round (SR, Figure 3 D) or pleomorphic (PL, Figure 3 E) synaptic vesicles and flat, symmetric postsynaptic densities were occasionally observed. Bushy cells of the rostral AVCN received a similar number of synaptic terminals per 10 µm^2^ compared to those of the caudal AVCN (p=0.114), but a greater percentage of rostral bushy cell perimeter was surrounded by terminals (p<0.001). The percentage of terminals displaying primary auditory nerve morphology was larger in caudal bushy cells compared to rostral cells (p=0.008). Non-primary nerve terminal profiles were more commonly observed contacting bushy cells of the rostral AVCN.

**Figure 4:**
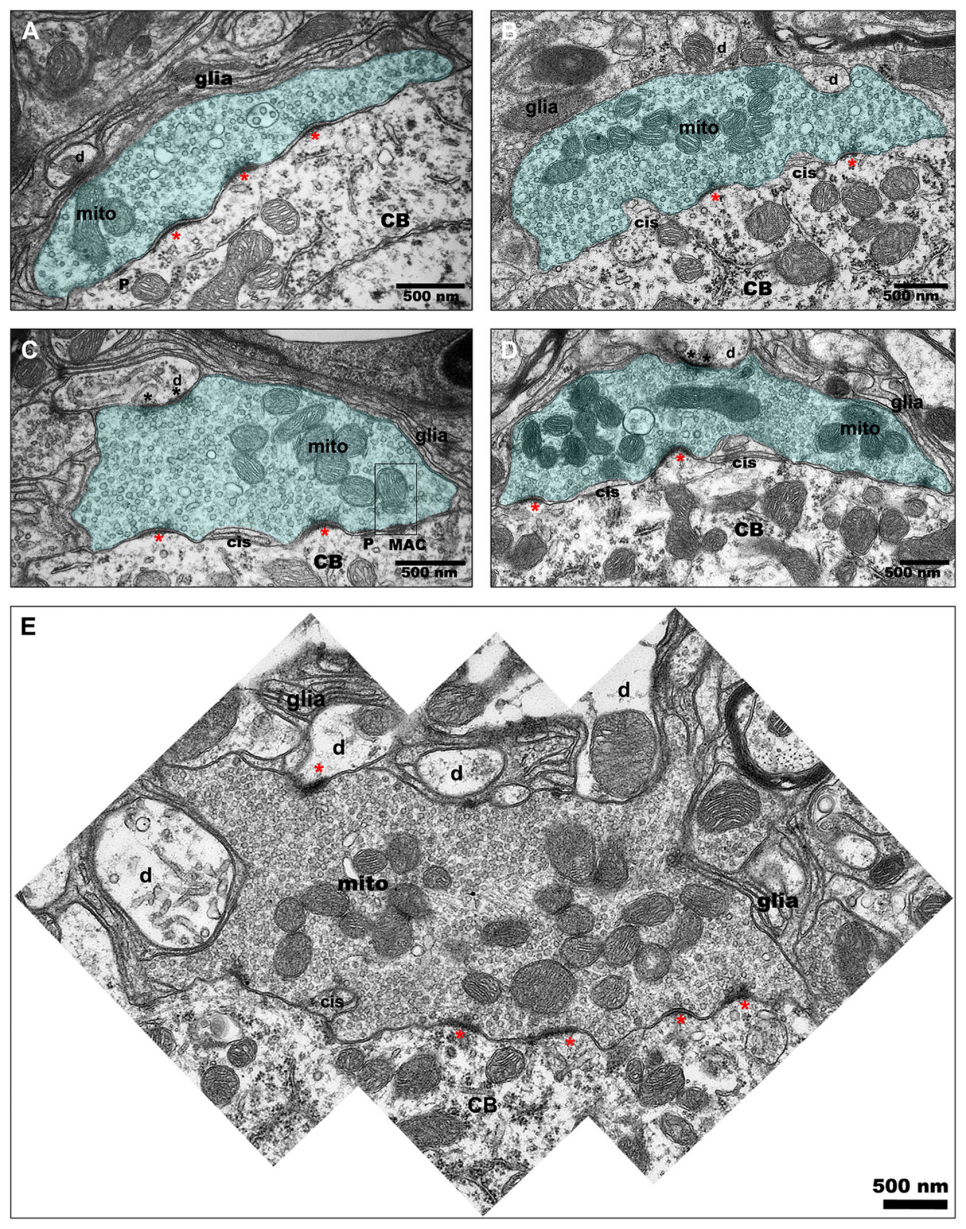
End bulb ultrastructure. Large auditory nerve terminals, or endbulbs, ensheathed in glial processes (glia) contact bushy cell bodies (CB) in both rostral AVCN and caudal AVCN (A–E). These terminals contain mitochondria clusters (mit) and many LR SVs associated with curved, asymmetric PSDs (single asterisks). Mitochondrial adherens complexes (MAC), puncta adherentia (puncta), and intermembranous cisternae (cis) are often observed. Endbulbs sometimes form synapses (double asterisks) with nearby dendritic processes (d) presumed to originate from nearby bushy cells. Occasionally, very large endbulbs are observed in proximity to numerous dendritic processes (E). Multiple release sites are common (A–D), but are not always separated by intermembranous cisternae (A, E).

A closer inspection of the ultrastructure of axosomatic primary auditory nerve profiles revealed many similar characteristics in rostral and caudal AVCN (Figure 3 F-G; Figure 4 A–E). A total of 49 primary terminal profiles from rostral AVCN bushy cells and 42 primary terminal profiles from caudal AVCN were analyzed. Primary terminals were large, replete with synaptic vesicles, and often displayed multiple curved PSDs. Intercellular cisternae, sometimes referred to as extended extracellular spaces, were often observed. Some of these cisternae contained small glial processes. Puncta adherentia and mitochondrion adherens complexes (MACs) were also observed in some profiles. Clusters of mitochondria within the terminals were frequent. One to six release sites consisting of a PSD, synaptic vesicles, and a synaptic cleft filled with a matrix of electron dense material were observed in end bulb profiles. Release sites were sometimes separated by extended extracellular spaces, which occasionally contained glial processes. Terminals were ensheathed in thin glial processes and occasionally made contact with dendrites. In some cases, the primary axosomatic terminals also formed synapses with these adjoining dendrites.

There was no statistical difference in the cross-sectional area of individual components of primary auditory nerve terminals (p=0.085), although there was a trend for larger profiles in rostral AVCN. PSD length (p=0.502), curvature (p=0.903), percent of terminal area occupied by mitochondria (p=0.396) were also not significantly different in rostral and caudal bushy cells. Mitochondrial cross-sectional areas were not evaluated. Previous studies have shown that some mitochondria near MACs form complex curved and branched structures [39]. Therefore, mitochondria profiles in a given section may be counted as multiple items when they actually originate from branches of the same mitochondrion. Primary terminals contacting rostral bushy cells contained more SVs per unit volume within 500 nm of the PSDs than caudal AVCN primary terminals (p=0.004).

### MNTB injections

**Figure 5:**
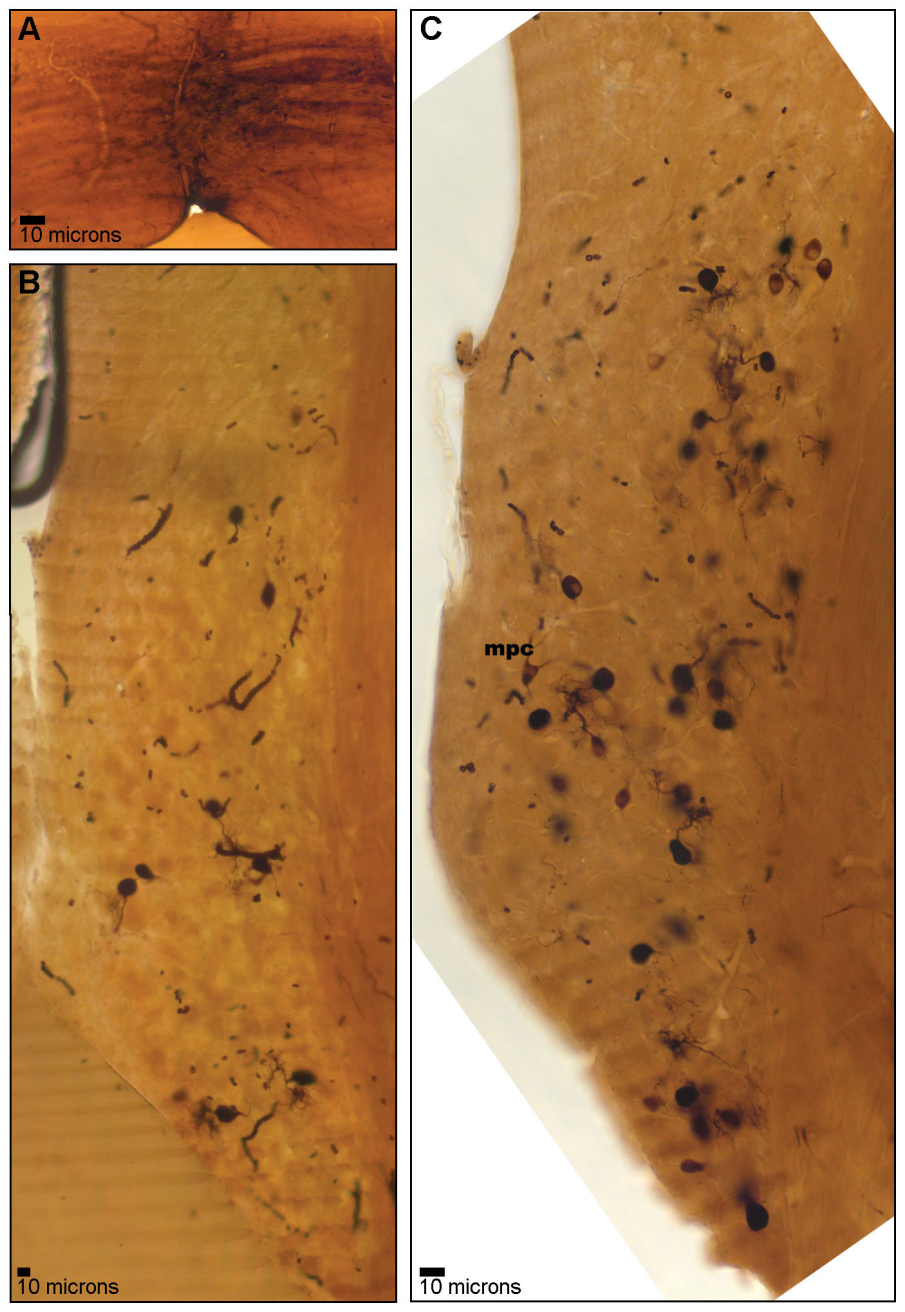
Bushy cells labeled by microinjection of tracer into the trapezoid body. Photomicrograph of trapezoid body injection site (A) and cells retrogradely labeled in caudal AVCN (B). Few cells were labeled in more rostral section (C).

**Figure 6:**
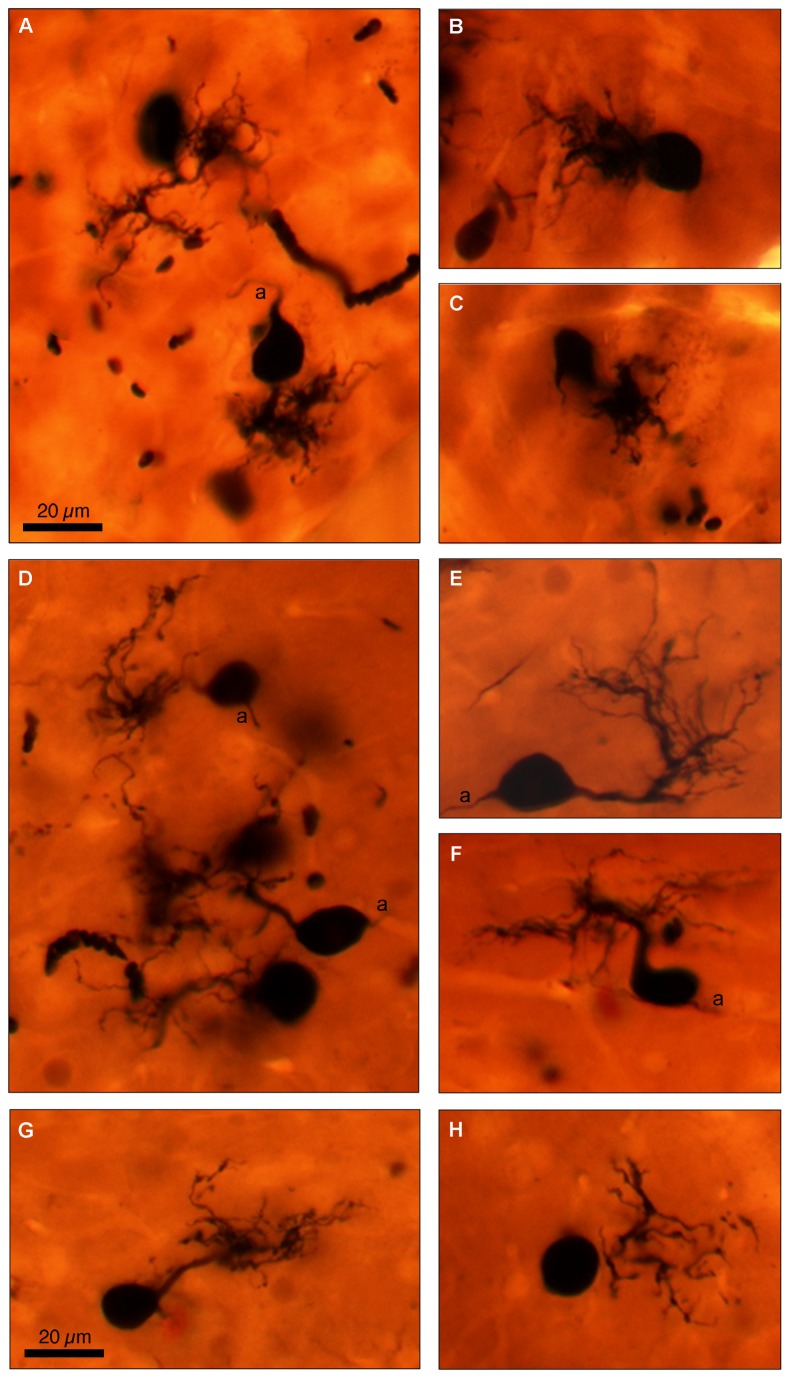
Examples of labeled bushy cells with varying dendritic morphology. (A–C) Cells with short, primary dendritic trunks capped with relatively compact dendritic branches. (D–H) Cells with longer dendritic trunks that bifurcate and send off diffuse, thin dendritic processes. Dendrites are not oriented in a particular direction. Scale bars apply to all panels.

A midline injection of BDA into the MNTB area (Figure 5 A) produced bilateral labeling of cells in AVCN. Labeled cells were predominantly localized in the core region of caudal AVCN (Figure 5 B), though several labeled cells were visible in rostral AVCN (Figure 5 C). The majority of labeled cells could be identified as bushy cells based on their characteristic round or elongated cell bodies and the single thick, bushy dendritic arbors (Figure 6 A–H). Nuclei, when visible, were located centrically or eccentrically within the cell body. Labeled bushy cells were often observed in clusters. A thin axon opposite the dendritic pole was visible in some cells (a; Figure 6 A, D, E, F). Labeled multipolar cells with irregularly shaped cell bodies and multiple dendrites were occasionally observed. Axons of T-multipolar cells course through the MNTB [40] and, thus, it is not surprising that some were labeled.

Bushy cells showed single, thick dendritic trunks emanating from one pole of the cell body. Two types of dendritic arbors emerged from the thick dendritic trunks. Some cells had short dendritic trunks capped with relatively compact dendritic tufts (Figure 6 A–C). Other cells had longer dendritic trunks that bifurcated and sent off diffuse, thin dendritic processes (Figure 6 D–H). Dendrites were not oriented in any particular direction within the AVCN, but some dendrites terminated in the region of other labeled bushy cells (Figure 6 D).

**Figure 7:**
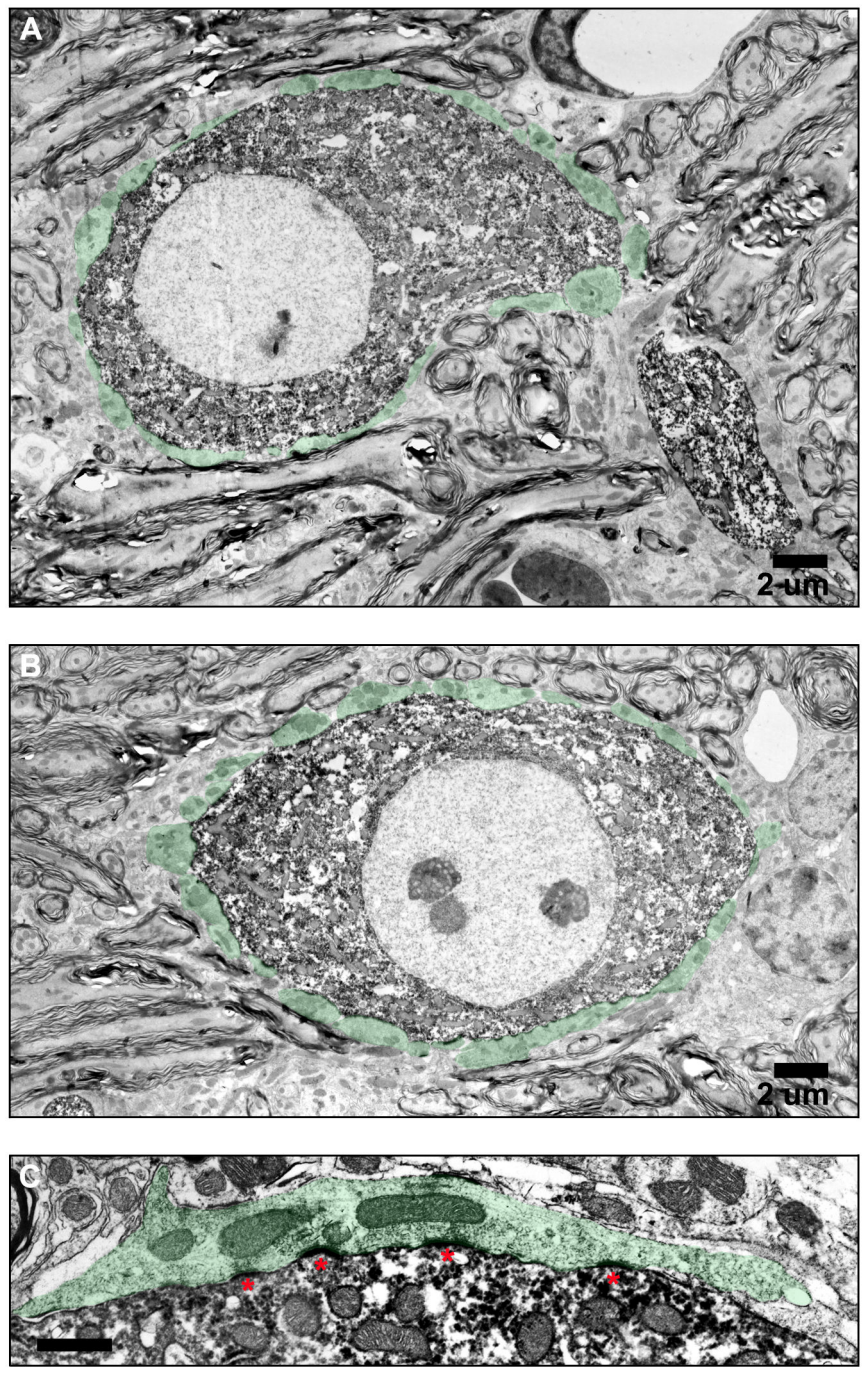
Electronmicrographs showing ultrastructural detail of labeled bushy cells. Synaptic terminals contacting two labeled bushy cells in caudal AVCN are highlighted in green (A, B). (C) High magnification image of a primary auditory nerve terminal forming a synapse with a labeled bushy cell. PSDs are marked with asterisks.

**Figure 8:**
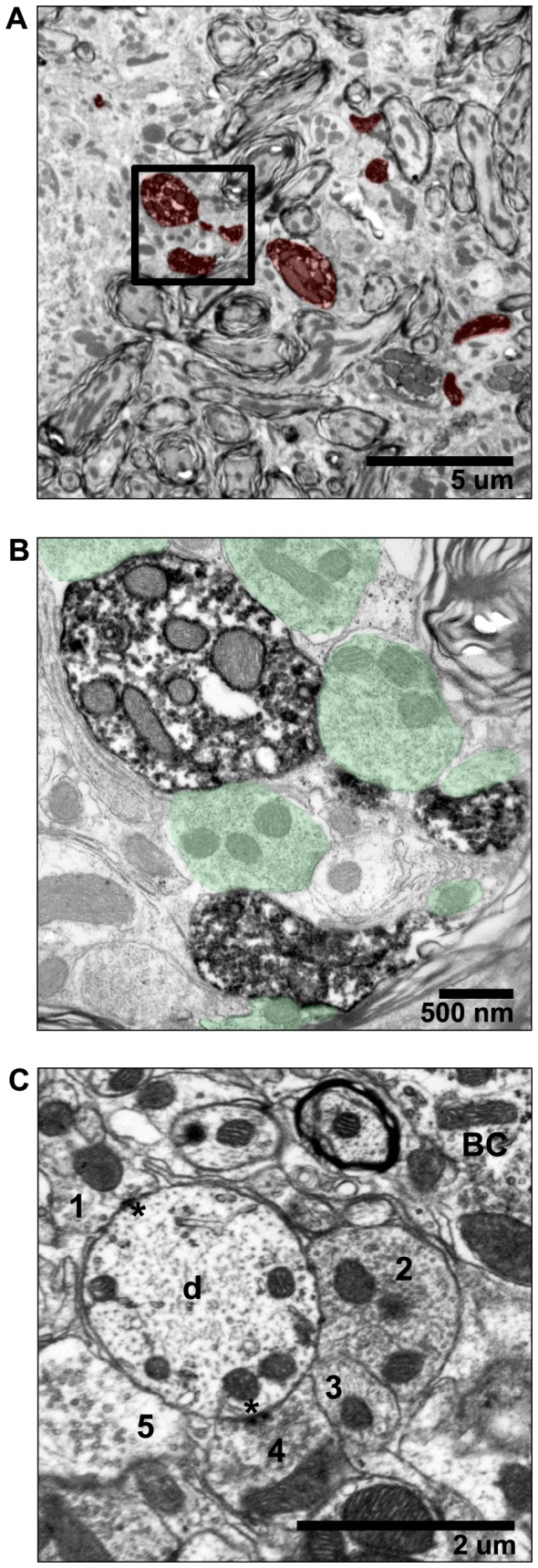
Electronmicrographs of bushy cell dendrites in cross section. (A) Multiple pieces of a labeled dendrite are visible in the neuropil surrounding bushy cells. (B) Bushy cell dendrites receive numerous bouton-like synaptic terminals (in green). This image shows terminals contacting the labeled dendrites in the boxed area in A. (C) Synaptic terminals (1–5) contacting an unlabeled dendrite (d) are shown so that ultrastructural details are visible. PSDs are marked with asterisks. Bushy cell (BC) body is indicated.

Electron microscopic imaging of labeled bushy cells revealed ultrastructural characteristics similar to those described in unlabeled bushy cells (Figure 7 A, B). Cell bodies contained a large, round centric or eccentric nucleus. The cytoplasm was replete with mitochondria, endoplasmic reticulum, and other organelles. A thick labeled dendritic trunk emerges from the cell in Figure 7 A. Labeled cell bodies were contacted by numerous large, glial-ensheathed terminals with curved PSDs (Figure 7 C). Ultrastructural features of the terminals were often obscured as a result of tissue processing artifacts.

Bushy cell dendrites received numerous synaptic terminals (Figure 8 A–C). Except when end bulb terminals formed synapses with nearby dendritic processes (Figure 4) presumed to represent distal dendrites from nearby bushy cells [31,33,41], the synaptic profiles were bouton-like with either large round or pleomorphic synaptic vesicles. These bouton terminals typically occurred in clusters and formed small PSDs (visible in Figure 8 C).

## Discussion

Mice have emerged as a model for hearing and the changes to auditory pathways that occur as a result of normal developmental processes, pathologies associated with hearing loss, genetic manipulation, aging, and manipulation of the acoustic environment. The study of binaural auditory pathways in mice becomes problematic because they rely primarily on interaural intensity cues for sound localization [42], congruous with insensitivity to low frequencies and small interaural distance that limit the efficacy of interaural timing cues.

The present anatomical analysis demonstrates that bushy cells of the mouse AVCN do not show the distinct regional differentiation as reported in cats [8]. Bushy cells did not show systematic differences in cell body size, shape, or nucleus eccentricity along the rostral-caudal axis of AVCN. However, rostral bushy cells contained more mitochondrial profiles than caudal bushy cells, which may reflect increased metabolic demands created by heightened levels of activity.

Light microscopic analysis of VGlut1-positive auditory nerve inputs to bushy cells did not reveal systematic differences along the rostral-caudal axis of AVCN; however, differences in inputs to bushy cell bodies were evident at the electron microscopic level. Rostral bushy cell somata received a larger proportion of non-primary synaptic inputs consisting of terminals with pleomorphic or small round synaptic vesicles compared to caudal bushy cells. These terminals with pleomorphic vesicles represent inhibitory inputs from a variety of sources including inhibitory neurons from dorsal cochlear nucleus and contralateral cochlear nucleus [43–49], and the lateral, medial, and ventral nuclei of the trapezoid body and superior paraolivary nucleus [50–53]. Terminals with small round vesicles represent excitatory inputs possibly originating from cholinergic neurons from the superior olivary complex and the pontomesencephalic tegmentum [54–56]. Serotonergic inputs from the dorsal and median raphe nuclei and noradrenergic inputs may also modulate bushy cell activity [57–61].

Rostral bushy cells also showed a greater overall proportion of inputs contacting the cell body surface when visualized with electron microscopy, many of which were non-primary terminals with pleomorphic vesicles presumed to represent inhibitory inputs of diverse origins. The larger proportion of primary auditory nerve contacts identified via electron microscopy in caudal bushy cells may reflect a greater degree of auditory nerve fiber convergence as reported for cat globular bushy cells [36]. Though the degree of auditory nerve fiber convergence onto bushy cells was not quantified in the present study, Cao and Oertel [23] estimated that cells with spherical bushy cell-like responses receive 1 to 2 auditory nerve fibers, whereas cells with globular bushy cell-like responses received 3 or more converging auditory nerve inputs.

Ultrastructural features of the auditory nerve inputs to rostral and caudal bushy cells were similar to that described for other mammalian species [31,33–36,38,41,62–70]. Mouse bushy cells received large auditory nerve endbulbs synapses and smaller bouton-like auditory nerve terminals encircling much of the cell body surface; they were positively labeled for VGlut1 immunohistochemical reaction product and contained large round synaptic vesicles. Auditory nerve terminals profiles had a light, clear cytoplasm, contained clusters of mitochondria, were ensheathed in glial processes, and associated with curved, asymmetric PSDs.

Large end bulb profiles often showed multiple release sites, which were sometimes separated by extended extracellular spaces. Variations in the distribution of release sites may account for differences in synaptic desensitization observed in brain slice preparations [71–73]. The presence of extended extracellular spaces, particularly those containing glial processes, may facilitate clearance of glutamate from the release site thereby constraining the temporal response [74]. On the other hand, release sites that are not isolated from one another could increase de-sensitization of the synapse and increase synaptic depression due to residual glutamate remaining in the synaptic cleft and spillover to adjacent sites. The regional differences observed in end bulb SV density and mitochondrial content may also reflect variation in synaptic activity.

Retrograde injection of neuronal tracer into the MNTB confirmed that this area receives projections from bushy cells in the caudal portion of AVCN. The dendritic arbors of labeled bushy cells were morphologically similar to those described in other species [10,21,22,64,75–80].

Proximal dendrites received numerous synaptic contacts in mice. In contrast to what has been reported in cats, distal dendritic segments also received numerous excitatory and inhibitory inputs in mice. It is unclear if the difference is species-related or due to the limited sampling with TEM. Distal dendritic processes were frequently observed in close proximity to bushy cell bodies. Similar to other species [31,33,36,41,64], endbulbs sometimes formed synapses with nearby dendrites presumed to originate from nearby bushy cells. Labeled dendritic processes near bushy cell bodies confirmed that at least some of these processes originate from other bushy cells (Figure 8). This occurrence has been interpreted as evidence of a network of bushy cell nests wherein the temporal precision may be enhanced. There is as yet no physiological evidence confirming the function of these nests.

In conclusion, mouse bushy cells are best differentiated by their inputs and axonal projections. The current results reiterate long standing questions about structure-function relationships in cochlear nucleus [21,22]. Prototypical examples of spherical and globular subtypes can be identified, but there is no clear regional boundary between subtypes within the AVCN. Mice have evolved hearing abilities that allow them to communicate at much higher frequencies than many other species used in hearing research; therefore, it is no surprise that the morphology of cells in the auditory pathways might differ because they are adapted to their specific processing needs. Recent studies of cochlear nucleus cell morphology have also identified possible species-specific variation in bushy cells and end bulb synapses [81–83]. The present report highlights the importance of conducting detailed anatomical investigations of auditory pathways in the particular species of interest because ultrastructural differences may account for physiological variations reported in different species.
